# Thiosulphate conversion in a methane and acetate fed membrane bioreactor

**DOI:** 10.1007/s11356-015-5344-3

**Published:** 2015-10-01

**Authors:** Diego A. Suarez-Zuluaga, Peer H. A. Timmers, Caroline M. Plugge, Alfons J. M. Stams, Cees J. N. Buisman, Jan Weijma

**Affiliations:** Sub-Department of Environmental Technology, Wageningen University, Bornse Weilanden 9, 6700 AA Wageningen, The Netherlands; Laboratory of Microbiology, Wageningen University, Dreijenplein 10, 6703 HB Wageningen, The Netherlands

**Keywords:** Anaerobic oxidation of methane, Thiosulphate disproportionation, Sulphur production, Green sulphur bacteria, Pyrosequencing, *Desulfocapsa*

## Abstract

The use of methane and acetate as electron donors for biological reduction of thiosulphate in a 5-L laboratory membrane bioreactor was studied and compared to disproportionation of thiosulphate as competing biological reaction. The reactor was operated for 454 days in semi-batch mode; 30 % of its liquid phase was removed and periodically replenished (days 77, 119, 166, 258, 312 and 385). Although the reactor was operated under conditions favourable to promote thiosulphate reduction coupled to methane oxidation, thiosulphate disproportionation was the dominant microbial process. Pyrosequencing analysis showed that the most abundant microorganisms in the bioreactor were phototrophic green sulphur bacteria (GSB) belonging to the family *Chlorobiaceae* and thiosulphate-disproportionating bacteria belonging to the genus *Desulfocapsa*. Even though the reactor system was surrounded with opaque plastic capable of filtering most of the light, the GSB used it to oxidize the hydrogen sulphide produced from thiosulphate disproportionation to elemental sulphur. Interrupting methane and acetate supply did not have any effect on the microbial processes taking place. The ultimate goal of our research was to develop a process that could be applied for thiosulphate and sulphate removal and biogenic sulphide formation for metal precipitation. Even though the system achieved in this study did not accomplish the targeted conversion using methane as electron donor, it does perform microbial conversions which allow to directly obtain elemental sulphur from thiosulphate.

**Electronic supplementary material:**

The online version of this article (doi:10.1007/s11356-015-5344-3) contains supplementary material, which is available to authorized users.

## Introduction

Sulphur compounds like sulphate, sulphite and thiosulphate are common constituents of aqueous effluents from chemical, mining and metallurgical industries. Biological treatment of these streams not only allows to remove the sulphur oxyanions from the aqueous effluents but also to recover sulphur which can be (re)used in chemical industry or as fungicide or fertilizer. Various industrial processes in which sulphur oxyanions are biologically reduced to sulphide have been developed and optimized (Stams et al. 2008). After reduction, the produced sulphide is either oxidized to elemental sulphur or used to precipitate metals. The latter allows to recover not only sulphur but also the used metals (Weijma et al. 2006).

Because industrial wastewaters often lack sufficient electron donor for biological reduction of the sulphur oxyanions, these need to be added from external sources. The most commonly added electron donors are ethanol and hydrogen (Meulepas et al. 2010). However, their use adds considerable operational and investment costs to the process (Meulepas et al. 2010). Industrial grade ethanol is fairly expensive for wastewater treatment purposes. Furthermore, although the feedstock for hydrogen production (methane) is relatively cheap, the required methane reformer is expensive. Using methane as direct electron donor for sulphate reduction through the microbiological process of anaerobic oxidation of methane (AOM) would allow to reduce operational costs (Meulepas et al. 2010). AOM is thought to rely on an obligate syntrophic relationship between anaerobic methanotrophic (ANME) archaea and sulphate reducing bacteria (Boetius et al. 2000; Hinrichs et al. 2000; Valentine and Reeburgh 2000). Because AOM consortia have doubling times between 1.2 and 7.5 months on methane and sulphate (Deusner et al. 2010; Girguis et al. 2005; Krüger et al. 2008; Meulepas et al. 2009a; Nauhaus et al. 2007; Zhang et al. 2010), economically feasible conversion rates in bioreactors have not been obtained yet (Suarez-Zuluaga et al. 2014). The highest specific AOM rate (370 μmol g_dry weight_^−1^ day^−1^) was obtained in a high-pressure continuous incubation (Deusner et al. 2010). Furthermore, the highest volumetric sulphate reduction rate (0.6 mmol L^−1^ day^−1^ or 286 μmol g_dry weight_^−1^ day^−1^) was reported by Meulepas et al. (2009a) in an 884-day experiment using a 2-L membrane bioreactor. However, these rates are still low considering that up to 100 times higher sulphate reduction rates can be achieved with hydrogen or ethanol.

To increase AOM rates for practical applications, we aimed to stimulate the growth of AOM consortia. Jagersma et al. (2012) found that incubating Eckernförde Bay sediment in the presence of thiosulphate and acetate increased the number of ANME microorganisms as compared to total Archaea. Furthermore, a higher standard Gibbs free energy can be obtained when using thiosulphate (reaction 1, Table 1) instead of sulphate (reaction 2, Table 1) for methane oxidation (−39.0 vs. −16 kJ mol^−1^ of CH_4_). It has been previously shown that this higher energy gain leads to higher AOM rates (Suarez-Zuluaga et al. 2014). However, in that study, part of the thiosulphate was disproportionated to sulphate and hydrogen sulphide (reaction 3, Table 1).

**Table 1:** Sulphate and thiosulphate reduction and thiosulphate disproportionation and their standard (Δ*G*°) and biological (Δ*G*°′) Gibbs free energy changes at pH 7.0 and the following concentrations: CH_4_ 0.9 mM, HCO_3_− 0.5 mM, SO_4_^2--^ 28 mM, S_2_O_3_^2--^ 14 mM and HS^−^ 0.5 mM

		Δ*G*°	Δ*G*°′
Reaction 1	CH_4_ + S_2_O_3_^2 −^ → HCO_3_^−^ + 2HS^−^ + H^+^	−39.0 kJ mol^−1^ CH_4_	−66.6 kJ mol^−1^ CH_4_
Reaction 2	CH_4_ + SO_4_^2 −^ → HCO_3_^−^ + HS^−^ + H_2_O	−16.5 kJ mol^−1^ CH_4_	−27.6 kJ mol^−1^ CH_4_
Reaction 3	S_2_O_3_^2 −^ + H_2_O → SO_4_^2 −^ + HS^−^ + H^+^	− 22.5 kJ mol^− 1^ S_2_O_3_^2 −^	−39.6 kJ mol^−1^ CH_4_

In this work, we aimed to rapidly enrich for microorganisms capable of performing AOM coupled to thiosulphate reduction. For this, we supplied acetate and thiosulphate as additional substrates besides methane and sulphate. This was done in a 5-L semi-batch membrane bioreactor inoculated with mixed sediments from Eckernförde Bay and Aarhus Bay. This bioreactor type not only allows complete biomass retention but also continuous removal of hydrogen sulphide. This is important as low concentrations of hydrogen sulphide inhibited AOM in Eckernförde Bay sediments or enrichments (Meulepas et al. 2009b). For the biomass used in this study, Suarez-Zuluaga et al. (2014) reported this concentration to be 5.4 mM.

## Materials and methods

### Source of microorganisms

The biomass used for inoculation originated from sediments of Aarhus Bay (Baltic Sea, Denmark) and Eckernförde Bay (Baltic Sea, Germany). The sampling site and method for the Eckernförde Bay sediment have been previously described by Meulepas et al. (2009a), and for the Aarhus Bay sediment, it was described in Suarez-Zuluaga et al. (2014). Both sediments were mixed in a ratio of 1:1, and 180 g L^−1^ (dry weight) was added to the reactor

### Medium

Synthetic seawater medium was prepared as described previously by Meulepas et al. (2009a). The initial sulphate concentration was 24 mM. Sodium thiosulphate (17 ± 2 mM) was added on days 0, 77, 120, 166, 258, 316 and 385, while acetate (0.6 ± 0.1 mM) was added on days 0, 57, 77, 82, 105, 120, 126 and 134. One mL L^−1^ of a 2 mM resazurin solution and 1 mL L^−1^ of a 0.9 M sodium sulphide solution were added to the medium during preparation.

### Reactor set-up

**Fig. 1:**
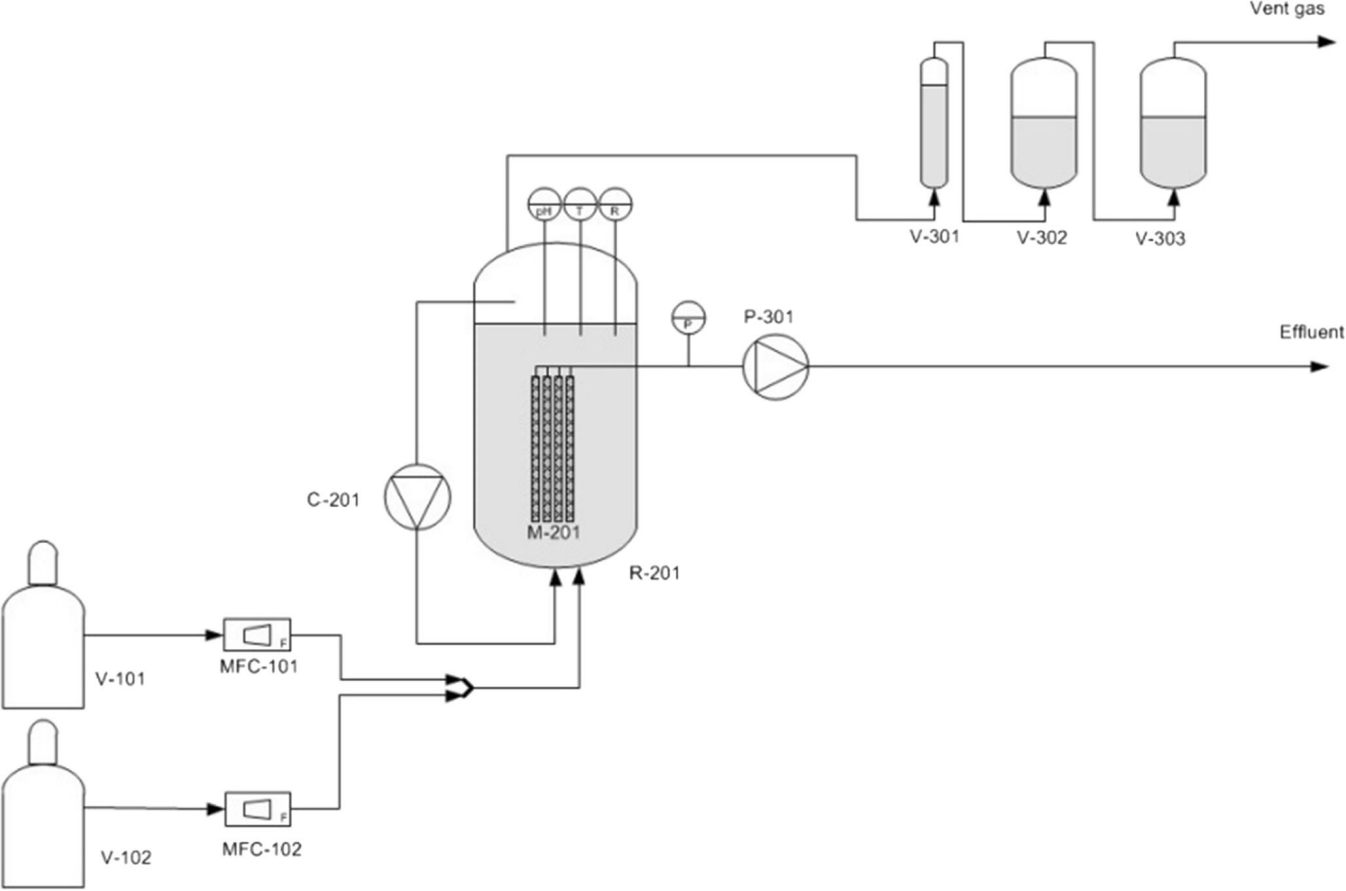
Reactor set-up. *V* vessels, *MFC* mass flow controllers, *P* pumps, *C* compressor, *R* reactor, *T* temperature, *R* redox sensors

The reactor set-up is shown in Fig. 1. The bioreactor (R-201) was a cylindrical glass vessel (height 50 cm, internal diameter 12 cm, total liquid volume 5 L), and it was operated as a fed-batch system, which means that the reactor was operated in batch mode and 30 % of the medium was replaced on days 77, 119, 166, 258, 312 and 385. Removal of the medium was done through four polysulphone membranes (M-201) (Triqua BV, Wageningen, The Netherlands), with a total effective surface area of 0.028 m^2^ and a filter pore size of 0.2 μm to achieve complete cell retention. The extraction of the media was performed using a peristaltic pump (P-301) (Watson Marlow 520U; Cornwall, UK). The transmembrane pressure was monitored with a pressure transmitter (PTX 1400; Druck, Leicester, UK). Redox potential was determined via a QR402X sensor (ProSense-Qis, Oosterhout, The Netherlands). The pH was monitored with a sulphide-resistant pH electrode (QP108X; ProSense-Qis, Oosterhout, The Netherlands), and it was controlled by manual addition of sodium hydroxide (0.2 M). A PT100 sensor was used to monitor the temperature. The reactor was equipped with a water jacket, and the temperature was controlled with a Julabo F25-ME water cooling system (Seelbach, Germany). The reactor was partially protected from day light by adding a blue screen to the reactor room. Light protection varied between 93 and 99.9 % depending on the light intensity available in the reactor surroundings. This variation was measured with a portable light meter (Li-Cor LI-250A) equipped with quantum sensor. Additionally on day 385, the reactor was covered with isolation foil to ensure a completely dark reactor environment.

Methane (99.9995 %; The Linde Group, Munich, Germany) from a gas cylinder (V-101) was continuously added to the reactor via a gas sparger at a flow rate of 2.2 L L_reactor_^−1^ day^−1^. This was done in order to supply methane to the microorganisms and to (partially) remove carbon dioxide and hydrogen sulphide via stripping and to prevent membrane fouling. Additionally, as a water lock (V-301) was connected to the bioreactor gas vent, a slight overpressure was maintained during the whole operation time. The methane flow was measured and controlled using a thermal mass flow controller (MFC-101) (5850E; Brooks, Veenendaal, The Netherlands). Nitrogen (V-102) (99.5 %; The Linde Group) was also added via a mass flow controller (MFC-102). Nitrogen sparging was done either to maintain anaerobic conditions in situations in which the reactor had to be opened (e.g. electrode calibration or sampling) or after day 312 when the methane feed was stopped.

The reactor was mixed twice a week via a laboratory compressor (P-201) (N 86 KT.18; KNF Laboport, Rowville, Australia).

A zinc chloride bottle of 1 M (V-302) was located and situated after the water lock from the beginning of the reactor operation. Another zinc chloride bottle (V-303) was added on day 77. As hydrogen sulphide was continuously stripped out of the reactor, V-302 and V-303 allowed to capture it as zinc sulphide.

Temperature, pH and redox where monitored in real time and data were logged into the computer using the LabView 7.1 software. Mass flow controllers were regulated through that software as well.

### Physicochemical analyses

To ensure reproducible and representative aqueous reactor samples, the samples were taken after at least 30 min of mixing with the P-201 compressor. Dry weight was determined according to standard methods (APHA 2005).

Acetate was measured by gas chromatography (Hewlett Packard 5890 Series II; Palo Alto, USA) according to Lindeboom et al. (2013). Methane, carbon dioxide and oxygen were also measured using gas chromatograph (GC-2010A; Shimadzu, Japan) according to Suarez-Zuluaga et al. (2014).

Sulphate and thiosulphate were quantified using a Dionex ICS-2100 ion chromatography system (Dionex, Salt Lake City, USA). Liquid samples of 5 μL were injected using a KOH gradient separated over a guard column (Dionex IonPac AS19, 4 × 50 mm) and an analytical column (Dionex IonPac AS19, 4 × 250 mm). The columns were operated at a temperature of 30 °C. A potassium hydroxide solution at a flow rate of 1.0 mL min^−1^ was used as an eluent. It was prepared online using the EG40 Eluent Generator (Dionex) equipped with a KOH cartridge (Dionex P/N 58900) and Milli-Q water. The KOH concentration gradient was programmed as follows: 10 mM from 0 to 10 min, followed by a 15-min gradient in which the concentration was linearly raised to 50 mM and kept there for 5 min. Finally, the KOH concentration was decreased to 10 mM during the last minute. An electrochemical detector (ECD) was used. Sample preparation prior to measurement consisted on centrifuging the sample at 10,000 rpm for 10 min. This was followed by mixing 0.2 mL of the liquid sample with 0.2 mL of the 1 M zinc acetate solution to capture hydrogen sulphide. After a second centrifugation to remove the captured zinc sulphide, 0.9 mL of the 1 M mannitol solution was mixed with 0.1 mL of the supernatant. Mannitol was added to stabilize sulphur compounds. The standards for sulphate had a concentration between 0.25 and 2 mM, while standards for thiosulphate with a concentration between 0.4 and 1.8 mM were used. Results were quantified using the Chromeleon 6.8 SR7 software.

Hydrogen sulphide was quantified colorimetrically in a reaction yielding methylene blue using standard kits (LCK 653) and a photo spectrometer (XION 500), both from Hach Lange (Düsseldorf, Germany). This method measures all the dissolved sulphide compounds (H_2_S, HS^−^ and S^2−^).

Total sulphur in the samples was analyzed by a combined microwave and inductively coupled plasma-optical emission spectroscopy (ICP-OES) (Vista-MPX CCD simultaneous; Varian, Inc., Palo Alto, USA). The method has been previously described by Hageman et al. (2013). The standard deviation in all measurements was ≤1.8 %.

Elemental sulphur was determined by high-performance liquid chromatography (HPLC). For this measurement, the sample was centrifuged prior to acetone extraction of the pellet. The exact conditions for sample preparation and measurement were described in Janssen et al. (1995).

Samples for scanning electron microscopy-energy-dispersive X-ray spectroscopy (SEM-EDX) measurements were washed three times with sulphur-free medium before analysis. Washing was done by centrifuging at 10,000 rpm for 10 min after which the supernatant was removed and the pellet was resuspended in sulphur-free medium. Repeating this procedure three times warranted that the only sulphur present in the sample corresponded to sulphur in solid form. The samples were placed on SEM sample holders by carbon adhesive tabs (EMS, Washington, USA) and subsequently coated with about 15 nm iridium. Samples were analyzed for morphology at 2 kV, 6 Pa, WD 5 mm at room temperature, in a field-emission scanning electron microscope (Magellan 400; FEI, Eindhoven, The Netherlands). EDX analyses were accomplished using the same electron microscope using a X-Max/AZtec X-ray analyzer (Oxford Instruments Analytical, High Wycombe, England) at an acceleration voltage of 15 kV, 200 Pa, WD 5 mm.

Several methods were used to detect polysulphide. The first one consisted on measuring the absorbance of the centrifuged sample at 285 nm as described by Teder (1967). Second, as described by Teder (1971) and Jørgensen et al. (1979), the zero-valent sulphur atoms in the polysulphide chains were separated by acidifying the sample from which other solids have been previously separated, in this case, by sedimentation. After this, elemental sulphur was measured as described above.

Batch tests with biomass taken from the reactor were done in order to determine if methanotrophic or methanogenic activity was observed. Biomass for these tests was taken on day 105 of the reactor operation. The tests were done in serum bottles which were made anoxic before adding the medium and the biomass. Cultures were incubated in the dark at 15 °C. The complete description of how this was done has been previously reported in Suarez-Zuluaga et al. (2014).

### Microbial community analysis

Genomic DNA was extracted using the Fast DNA Kit for Soil (MP Biomedicals, OH) according to the manufacturer’s protocol with two 45-s beat beating steps using a FastPrep Instrument (MP Biomedicals). Quantity and quality was checked using NanoDrop® ND-2000 (Thermo Scientific, Wilmington, DE).

Extracted DNA from the sampling point at 105 days was used for archaeal clone library construction. To amplify the almost full-length archaeal 16S ribosomal RNA (rRNA) genes for cloning, primers A109f (Grosskopf et al. 1998) and 1492R (Lane 1991) were used (primers are given in Table S1). PCR amplification was done with the GoTaq Polymerase kit (Promega, Madison, WI) using a G-Storm cycler (G-Storm, Essex, UK) with a pre-denaturing step of 120 s at 95 °C followed by 35 cycles of 95 °C for 30 s, 52 °C for 40 s and 72 °C for 90 s. Lastly, a post-elongation step of 5 min at 72 °C was done. PCR products were pooled and purified using the PCR Clean & Concentrator kit (Zymo Research Corporation, Irvine, CA), and product size was checked on a 1.5 % agarose gel. DNA quantification was done using NanoDrop® ND-2000 (Thermo Scientific, Wilmington, DE). Pure products were ligated into a pGEM-T Easy plasmid vector (pGEM-T Easy Vector System I; Promega, Madison, WI) and transformed into *Escherichia coli* XL1-Blue competent cells (Stratagene/Agilent Technologies, Santa Clara, CA). Both ligation and transformation were performed according to the manufacturer’s instructions. Nucleotide sequence data reported are available in the European Nucleotide databases under the accession nos. LN626804–LN626890.

Reactor samples for real-time quantitative PCR (qPCR) were taken after 15 days and after 90 days of reactor operation. Extracted DNA was purified using the OneStep PCR inhibitor removal kit (Zymo Research Corporation, Irvine, CA). The DNA concentration was determined with NanoDrop (Thermo Fisher Scientific, MA). Amplifications were done in triplicate in a Bio-Rad CFX96™ system (Bio-Rad Laboratories, Hercules, CA) in a final volume of 25 μL using iTaq Universal SYBR Green Supermix (Bio-Rad Laboratories), 5 ng of template DNA and primers with optimal concentrations and annealing temperatures for highest efficiency and specificity (Table S2), all according to the manufacturer’s recommendations. Triplicate standard curves were obtained with 10-fold serial dilutions ranged from 2 × 10^5^ to 2 × 10^−2^ copies per μL of plasmids containing 16S rRNA archaeal inserts of ANME-1a and ANME-2a/b. The efficiency of the reactions was up to 91 %, and the *R*^2^ of the real-time qPCR standard curves obtained was up to 0.997. All used primers were extensively tested for specificity with cloned archaeal inserts of ANME-1a and ANME-2a/b and with genomic DNA of *Methanosarcina mazei* TMA (DSM-9195) and *Desulfovibrio* sp. G11 (DSM-7057). PCR conditions consisted of a pre-denaturing step for 5 min at 95 °C, followed by 5 touch-down cycles of 95 °C for 30 s, annealing at 65 °C for 30 s with a decrement per cycle to reach the optimized annealing temperature, and extension at 72 °C (times are shown in Table S2). This was followed by 40 cycles of denaturing at 95 °C for 20 s, 30 s of annealing (temperatures are shown in Table S2) and extension at 72 °C (times are shown in Table S2). PCR products were checked for specificity by melting curve analysis (72–95 °C) after each amplification step and gel electrophoresis. Quantification of specific archaeal and bacterial groups was expressed as the number of copies per gram dry weight and relative to the amount of total archaeal products.

Extracted DNA from the sampling point at 105 days was used for bacterial 16S rRNA gene pyrosequencing. Barcoded amplification of the V1–V2 region of the 16S rRNA gene was done using forward primer 27F-DegS (van den Bogert et al. 2011) that was extended with titanium adapter A and an eight-base sample-specific barcode (Hamady et al. 2008) at the 5′ end and an equimolar mix of reverse primers 338R-I and 338R-II (Daims et al. 1999) that were appended with titanium adapter B at the 5′ end. All primers used are given in Table S1. PCR amplification was performed in a SensoQuest labcycler (SensoQuest GmbH, Goettingen, Germany) in a total volume of 100 μL containing 2 μL of DNA (20 ng μL^−1^), 500 nM of barcoded forward primer and reverse primer mix (Biolegio BV, Nijmegen, The Netherlands), 2 U of Phusion Hot Start II High-Fidelity DNA polymerase (Finnzymes, Vantaa, Finland), 20 μL of 5× HF buffer, 2 μL of PCR grade nucleotide mix (Roche, Diagnostics GmbH, Mannheim, Germany) and 65 μL of nuclease-free sterile water. PCR amplification conditions were as follows: a pre-denaturing step of 3 min at 98 °C followed by 30 cycles of 98 °C for 10 s, 56 °C for 20 s and 72 °C for 20 s. Lastly, a post-elongation step of 10 min at 72 °C was done. PCR products were loaded on a 1 % (*v*/*v*) agarose gel containing 1× SYBR Safe (Invitrogen) to check the PCR product size and were purified using a GeneJet PCR purification kit (Thermo Fisher Scientific). The concentration was determined using the Qubit 2.0 fluorometer (Thermo Fisher Scientific). All the samples for pyrosequencing were afterwards pooled in equimolar amounts. Pooled samples were loaded on a 1 % (*v*/*v*) agarose gel containing 1× SYBR Safe (Invitrogen), and bands of the correct size were excised and purified with the GeneJet Gel Extraction kit (Thermo Fisher Scientific) using 25 μL elution buffer for collecting the DNA. Pooled samples were quantified using the Qubit 2.0 fluorometer (Thermo Fisher Scientific) and submitted for pyrosequencing on the 454 Life Sciences GS-FLX platform by titanium sequencing chemistry (GATC Biotech, Germany).

The pyrosequencing data were analyzed with a workflow based on Quantitative Insights Into Microbial Ecology (QIIME) v1.2 (Caporaso et al. 2010), and reads were filtered for chimeric sequences using the USEARCH algorithm (Edgar 2010). Operational taxonomic unit (OTU) clustering was performed with settings as recommended in the QIIME newsletter in December 17, 2010 (http://qiime.wordpress.com/2010/12/17/new-default-parameters-for-uclust-otu-pickers/), using an identity threshold of 97 %. Diversity metrics were calculated as implemented in QIIME 1.2. The SILVA reference database was used for taxonomic classification (Quast et al. (2013)). After picking representative OTUs, we quantified the absolute and relative amount of reads of every OTU and only considered OTUs that are highly abundant (>100 reads/OTU).

## Results and discussion

### Reactor operation until day 152

**Fig. 2:**
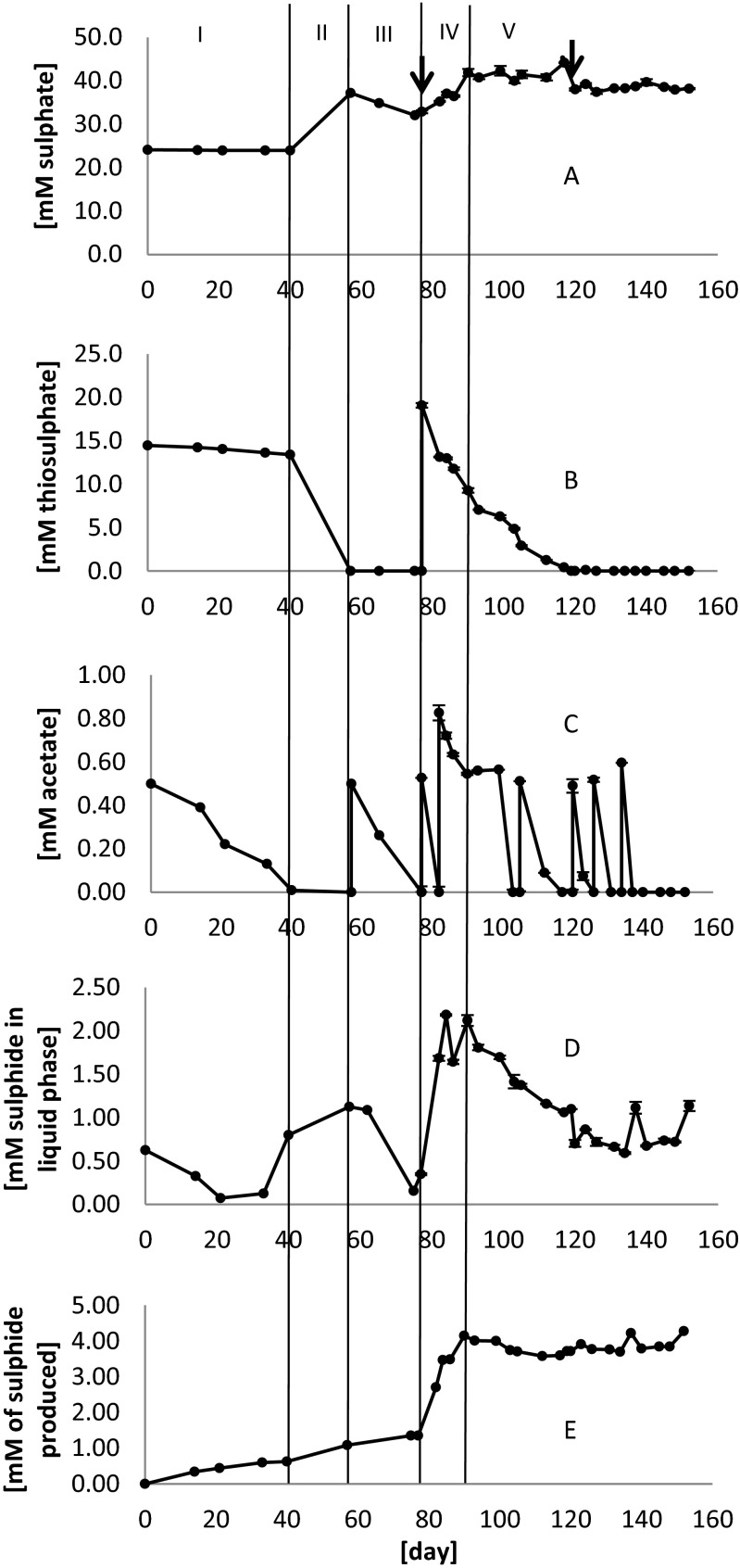
Measured compound concentrations and net sulphide production. *A* Sulphate. *B* Thiosulphate. *C* Acetate. *D* Sulphide. *E* Net sulphide production. The *arrows* indicate the days in which medium was partially replaced. *Error bars* represent the standard deviation of duplicate measurements. The *vertical lines* indicate the limit of the time phases that are used to explain the figure. *I* days 0–40, *II* days 40–57, *III* days 57–77, *IV* days 77–90, *V* days 90–152

The concentrations of sulphate (Fig. 2(A)), thiosulphate (Fig. 2(B)), acetate (Fig. 2(C)) and sulphide (Fig. 2(D)) in the reactor up to day 152 are presented. Additionally, Fig. 2(E) shows the net sulphide production, which accounts not only for the sulphide present in the reactor but also for the sulphide that is stripped out and captured as zinc sulphide. The pH of the reactor was kept around 7.3 (±0.4) during the whole experiment (data not shown). The redox potential was −375 mV (±75 mV) (data not shown).

During phase I (Fig. 2), the initial 0.5 mM of added acetate was consumed while the thiosulphate concentration slightly decreased (1.1 mM at a rate of 0.028 mmol L^−1^ day^−1^). This could have been either due to reduction with acetate (reaction 4) and organic compounds that might be present in the sediments or due to disproportionation (reaction 3). However, as the sulphate concentration did not change, there was no clear indication for the latter. Furthermore, acetate might have been used as a carbon source for anabolic use.

Reaction 4: CH_3_CO_2_^−^ + S_2_O_3_^2 −^ + H_2_O → 2HCO_3_^−^ + 2HS^−^ + H^+^

After the depletion of acetate on day 40 (phase II, Fig. 2), the thiosulphate consumption rate increased at least 29-fold (13.4 mM consumed in 17 days, 0.79 mmol L^−1^ day^−1^). Concomitantly, 13.2 mM of sulphate was formed, resulting in a thiosulphate decrease-to-sulphate increase ratio close to 1:1. This indicated that thiosulphate was disproportionated according to reaction 3. Apparently, as shown before by Pikaar et al. (2013), thiosulphate-reducing bacteria shifted to disproportionation after acetate was depleted. However, there was only 0.45 mM of sulphide formed and it was unlikely that it escaped through the gas vent. This suggested than an unknown sulphur compound was being formed from thiosulphate.

Acetate (0.50 mM) was again added on day 57, and it was consumed by day 77 (phase III, Fig. 2). During this 20-day period, in which thiosulphate was not present in the reactor, 4.3 mM of sulphate was consumed. According to reaction 5, and assuming that the acetate was only used for metabolic purposes, the consumed acetate could only account for about 12 % of the sulphate removal.

Reaction 5: CH_3_CO_2_^−^ + SO_4_^2 −^ → 2HCO_3_^−^ + HS^−^

As it can be expected that the inoculum was depleted of easily biodegradable electron donors by this time, this result pointed to AOM (reaction 2). However, only 0.27 mM of sulphide production was measured (Fig. 2(E)), which accounts for only about 6 % of the sulphur species removed as sulphate, indicating again that an unidentified sulphur compound had formed. The nature of this compound will be looked into in the following sections.

On day 77, 30 % of the medium was replaced. On this day, also thiosulphate (19.1 mM) and acetate (0.53 mM) were added. From that moment and up to day 90 (phase IV, Fig. 2), 9.8 mM of thiosulphate was steadily consumed, while 2.8 mM of sulphide and 9 mM of sulphate were produced. The ratio of −1:+0.9 for depleted thiosulphate to formed sulphate indicates that at least 90 % of the depleted thiosulphate was disproportionated. However, the thiosulphate depleted-to-sulphide formed ratio of −1:+0.3 indicates a maximum of only 30 % of sulphide was produced. In addition, of the 19.6 mM of sulphur removed as thiosulphate, only 11.8 mM of sulphur species was recovered as sulphate and sulphide, indicating again the formation of an unidentified sulphur compound(s).

Finally, phase V (days 90 to 152) was characterized by thiosulphate consumption (9.3 mM) with barely any net sulphate or sulphide production (2.4 and 0.1 mM, respectively). This indicates that both sulphate and sulphide were being removed from the system. Sulphate removal was possibly done through sulphate reduction, and to understand the mechanism of sulphide removal, several additional analyses were carried out.

Solid sulphur species were measured by total sulphur analyses (ICP, performed on day 99) of an untreated reactor sample (60.2 mM of S ± 1.8 %) and a centrifuged reactor sample (54.7 mM of S ± 1.8 %). Furthermore, polysulphide in the supernatant was detected by spectrophotometry and HPLC analysis. As the used sulphide measurement only accounts for one sulphur atom per polysulphide chain, not only solid sulphur but also polysulphide might account for the sulphur gap in the mass balance. However, results show that the total sulphur in the supernatant corresponded fairly well with the sum of sulphide, sulphate and thiosulphate concentrations (Table 2, day 99). Therefore, polysulphide and other unidentified soluble sulphur compounds could only contribute slightly, if at all, to the sulphur balance.

**Table 2:** Soluble sulphur compound concentrations (day 99)

	[mM sulphur]
H_2_S	1.7 ± 0.04
SO_4_	42.3 ± 2.2
S_2_O_3_	12.6 ± 0.4
Total	56.6 ± 2.6

**Fig. 3:**
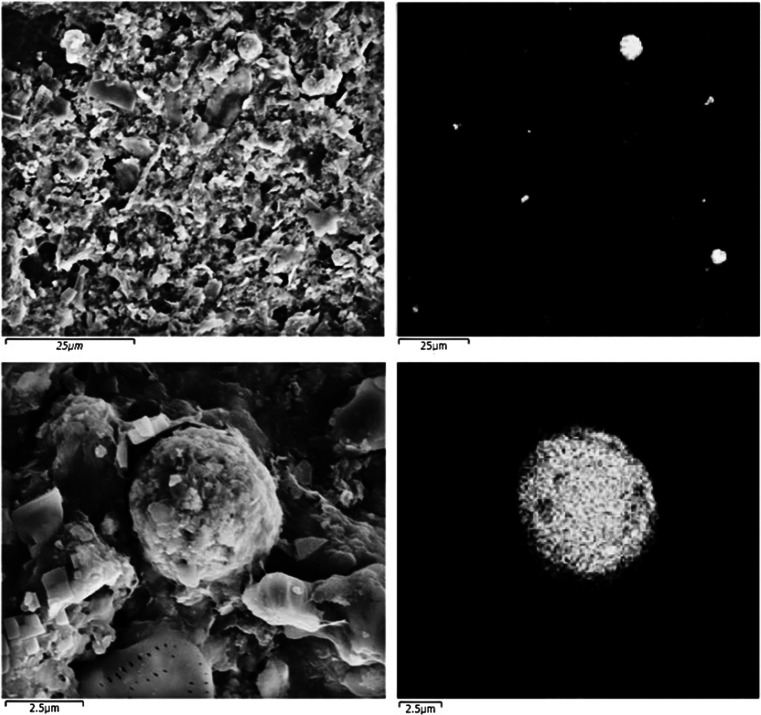
SEM-EDX pictures of the reactor contents. The *left side pictures* show to the original SEM image, while the *clear areas in the right side images* represent the sulphur that was identified in the samples

Green aggregates were observed under the microscope from around day 99 onwards. Furthermore, the presence of elemental sulphur in the pellet of the reactor sample and the green aggregates was confirmed by HPLC measurements (data no shown) and SEM-EDX analysis (Fig. 3).

The results obtained did not show evidence of AOM taking place. Instead, the dominant microbial processes that seem to be taking place in the reactor were thiosulphate disproportionation into sulphate and sulphide, followed by sulphide oxidation into elemental sulphur. Microbial community analyses were performed in order to obtain a better understanding of the conversions occurring in the reactor.

### Microbial community analysis

Biomass samples taken from the reactor at day 105 were subjected to 16S rRNA pyrosequencing for bacterial community analysis and 16S rRNA gene cloning for archaeal community analysis.

**Fig. 4:**
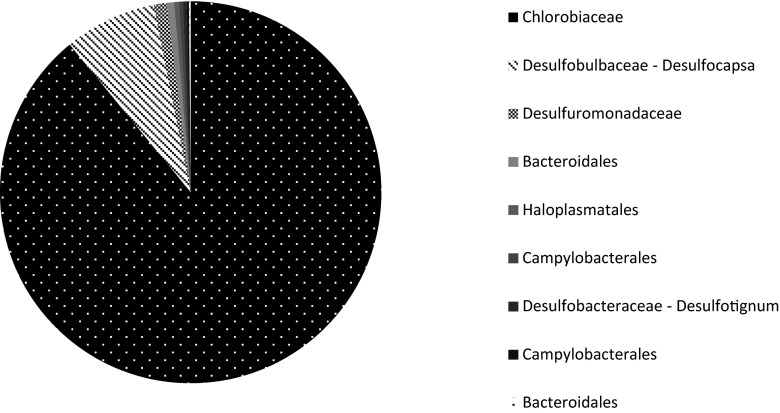
Bacterial 16S rRNA gene pyrosequencing results of the reactor inoculated with the Eckernförde Bay sediment and the Arhus Bay sediment. The sample was taken at 105 days of incubation. Relative abundance of only major groups with >100 reads/OTU at the order level is shown

Pyrosequencing results show that the most abundant group of microorganisms belonged to the *Chlorobiaceae* family (Fig. 4) of which all sequences show at least 98 % similarity to *Prosthecochloris aestuarii* (Gorlenko 1970). These green sulphur bacteria (GSB) comprised 86 % of all reads retrieved from the reactor. GSB are strictly anaerobic microorganisms that assimilate carbon dioxide only in the presence of light while oxidizing sulphide to elemental sulphur (reaction 6) (Gorlenko 1970; Frigaard and Dahl 2009). GSB use bacteriochlorophyll *c* for this phototrophic sulphide oxidation (Gorlenko 1970), which explains the green aggregates in the reactor. Furthermore, GSB are highly efficient photoautotrophic microorganisms; they have been found at 100 m depth in the Black Sea (Manske et al. 2005) and at a deep-sea hydrothermal vent (Beatty et al. 2005), explaining why GSB were able to grow in the reactor even though it was kept in a nearly-dark environment.

Reaction 6: $2{\mathrm{H}\mathrm{S}}^{-}+{\mathrm{CO}}_2\overset{\mathrm{light}}{\to }2{\mathrm{S}}^{\mathrm{o}}+{\mathrm{CH}}_2\mathrm{O}\left(\mathrm{carbohydrate}\right)+{\mathrm{H}}_2\mathrm{O}$ (Tang et al. 2009)

Bioreactor studies in which AOM was performed, such as the one of Meulepas et al. (2009a), did not report presence of GSB. However, that reactor was covered with opaque plastic in order to protect it from light and to avoid the interference of phototrophic microorganisms. Our results indicate that partially filtering light is not enough and it is of extreme importance to keep bioreactors targeted to cultivate slow-growing microorganisms such as those involved in AOM in complete darkness.

The second most abundant group detected by the pyrosequencing analysis shows a 100 % similarity with *Desulfocapsa sulfexigens* strain DSM 10523. *Desulfocapsa* spp. are known to be capable of performing thiosulphate disproportionation (Finster et al. 1998). Moreover, the sulphide removal by GSB creates a thermodynamically favourable condition for *Desulfocapsa* (Finster et al. 1998) which can produce more sulphide for the GSB to consume, creating a mutualistic symbiotic relationship. Furthermore, acetate has been reported to stimulate growth of not only *Desulfocapsa* (Janssen et al. 1996) but, if sulphide and carbon dioxide are present, also GSB (Gorlenko 1970). Therefore, as *D. sulfexigens* has not been previously associated with AOM, the presence of thiosulphate and acetate and the continuous sulphide removal were the most probable reason for its enrichment.

Finally, also, sequences of sulphate-reducing bacteria were found. They belong to the family of *Desulfuromonadaceae*, with 97–100 % similarity to *Desulfuromusa bakii* strain Gyprop and sequences related to *Desulfotignum toluenicum* strain H3 (99 % similarity) (Ommedal and Torsvik 2007). The number of reads of these sulphate- reducing bacteria was low (<1.5 %) and sulphate reduction was probably fuelled by acetate and/or organic compounds released by cell debris.

**Fig. 5:**
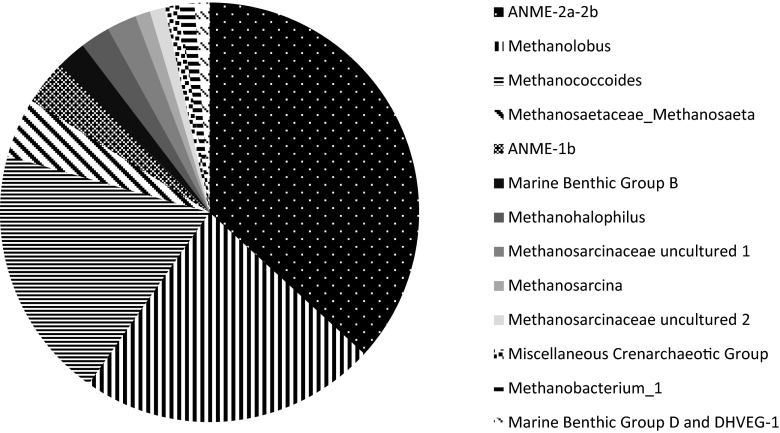
Archaeal 16S rRNA gene clone library results of the reactor inoculated with the Eckernförde Bay sediment and the Arhus Bay sediment. The sample was taken at 105 days of incubation

Archaeal clone library analysis revealed low diversity of archaeal sequences (Fig. 5), and the most abundant sequences found belonged to anaerobic methanotrophic Archaea from the ANME-2a/b subtype (37 % of sequences). Other anaerobic methanotrophic Archaea found that belonged to ANME-1b comprised only a minor fraction of the archaeal community (3.4 %). These results suggest that the conditions provided to the reactor did promote enrichment of ANME-2a/b microorganisms. Quantitative PCR was therefore performed to determine if these methanotrophic ANME-2a/b Archaea were actively growing.

**Fig. 6:**
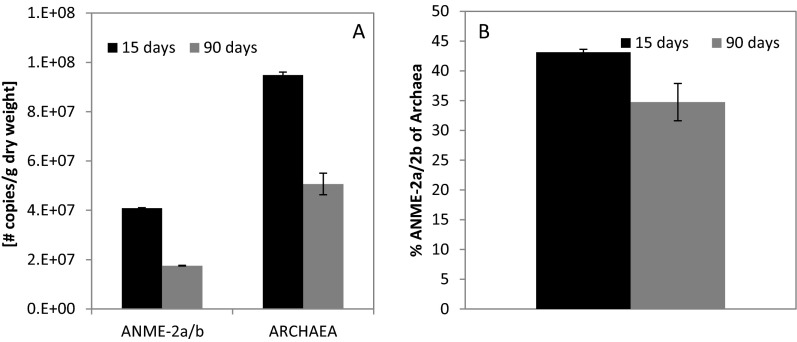
*Left* qPCR results of ANME-2a-specific primer and general archaeal primer copies per gram. *Right* ratio of ANME-2a/b over Archaea. The samples were taken at 15 and 90 days. Standard deviation for triplicate measurements

Reactor samples for qPCR were taken after 15 days and after 90 days of reactor operation. Results show that ANME-2a/b did not increase in absolute copies per gram dry weight nor in relative amount to total *Archaea* during reactor operation between these days (Fig. 6). ANME-2a/b and total archaeal numbers decreased during reactor run, which is consistent with the sulphide oxidation and thiosulphate disproportionation as dominant processes, while sulphate reduction was barely observed. The relative abundance of ANME-2a/b remained stable while the absolute numbers of ANME-2a/b decreased. This could be due to the constant feeding of methane and sulphate, which could sustain some viability of anaerobic methanotrophs. This is consistent with the clone library results that the ANME-2a/b was the most abundant sequence found. However, these results provide evidence that net growth of ANME Archaea was not achieved. Other Archaea that showed relatively high presence in the clone library belonged to *Methanolobus* (23 %) and *Methanococcoides* (20 %), which are both methylotrophic methanogens commonly found in marine sediments. However, methanogenic activity was not observed in batch tests made with biomass taken from the reactor, meaning that these Archaea were already present in the inoculum.

### Overall reactor operation

On day 152, an ICP measurement was performed. Not only was the sulphur concentration in the total sample (44.4 mM of S ± 1.8 %), and its supernatant measured (40.0 mM S ± 1.8 %), but also its pellet (5.4 mM S ± 1.8 %) and the pellet from the green reactor content (GSB) (2.0 mM ± 1.8 %). A sulphur mass balance for the reactor operation until day 152 was obtained using the results from this ICP analysis plus the total change in concentration of the measured soluble sulphur compounds (Table 3). The sulphur in the polysulphide chains (0.7 mM) was estimated from the difference between the sulphur measured in the supernatant of the total sample by ICP analysis and the total sulphur obtained by adding the sulphate (38.2 mM), sulphide (1.1 mM) and thiosulphate (0 mM) measured on day 152. The sulphur balance closes on about 90 % (33.2 mM of S consumed vs. 29.8 mM of S produced). The missing 10 % (3.4 mM) can be accounted to solid sulphur settled in the bottom of the reactor.

**Table 3:** Sulphur balance for the reactor operation until day 152

	[mM sulphur]
Sulphate	19.4
Thiosulphate	−33.2
Sulphide	4.3
Zero-valent sulphur in polysulphide	0.7
Sulphur in GSB	2.0
Solid sulphur forms	3.4
Non-measured sulphur	−3.4

The standard deviation of the measurements of the zero-valent sulphur, sulphur in GSB and solid sulphur forms is ±1.8 %, and the standard deviation of sulphate, thiosulphate and sulphide measurements can be observed in Fig. 2

Methane continued to be fed to the bioreactor, and 30 % of the medium was replenished on day 166. However, up to day 257, sulphate was not reduced and AOM activity was not demonstrated. Furthermore, neither evidence of methanotrophic nor methanogenic activity was found in batch experiments performed with biomass taken from the reactor. Additionally, as mention in the “Microbial community analysis” section, qPCR analysis did not show an increase in either ANME-1 or ANME-2 methanotrophic Archaea. These results indicate that AOM was not enhanced in our study. Our reactor was designed and operated under conditions thought to be favourable to increase methanotrophic activity (pH 7.3, redox −375, temperature 15 °C). However, neither this nor sulphate consumption was observed. Instead, growth of GSB and *Desulfocapsa* in a presumably symbiotic relationship took place. Perhaps, if complete darkness had been provided to the reactor and sulphide concentration had been kept closer to zero, GSB would not have been able to grow and this might have avoided that *Desulfocapsa* and GSB outgrow the microorganisms capable of performing AOM. Another reason might be that unlike the reactor from Meulepas et al. (2009a) and other reactors in which AOM has been increased, our system was thoroughly mixed and this might have created shear stress which the ANME could not stand. Finally, although the sulphide concentration was kept below inhibition levels, the lack of continuous mixing might have created zones in the reactor with high sulphide concentrations hindering the growth of ANME Archaea.

**Fig. 7:**
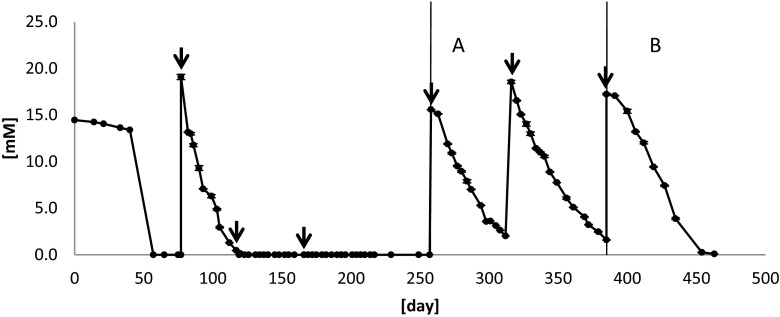
Thiosulphate concentration in liquid phase. Acetate was not added in either phase A or B. Methane feeding was interrupted on day 316. The reactor was completely covered from light on day 385. The *arrows* indicate the days in which the medium was replaced. *Error bars* represent the standard deviation of duplicate measurements. The *vertical lines* indicate the time phases that are used to explain the figure. *A* days 258–385, *B* days 385–454

To further analyze the contribution of the microorganisms found in the pyrosequencing analysis, the reactor was subjected to two additional operational phases; 30 % of the medium was replaced at the beginning of each. Results are presented in Fig. 7, and Table 4 presents the mass balance.

**Table 4:** Sulphur mass balance in the liquid phase between days 258 and 385. Acetate was not added in either phase A or B of Fig. 7. Methane feeding was interrupted on day 316. The reactor was completely covered from light on day 385

	Days 258–385 (nearly dark)	Days 385–454 (dark)
	Delta compound [mM]	Delta compound [mM]
Thiosulphate	−30.5	−17.0
Sulphate	29.5 (0.97)	17.5 (1.03)
Sulphide	8.1 (0.26)	7.5 (0.44)

The numbers between parentheses correspond to the fraction of sulphate and sulphide produced from the consumed thiosulphate which, stoichiometrically, should be 1.0 (reaction 3)

In the first period (days 258–385) (phase A, Fig. 7), thiosulphate was added and methane was initially fed, but no acetate was supplemented. Thiosulphate was added again on day 316. However, on this day, the methane feeding was stopped until the end of the reactor run. As expected, ending the methane and acetate addition did not have an influence of the observed reactions and the results were similar to those previously discussed (“Reactor operation until day 152” section). Thiosulphate disproportionation did occur. However, as the GSB continued producing sulphur from sulphide, the latter did not match the stoichiometry (Table 4).

**Fig. 8:**
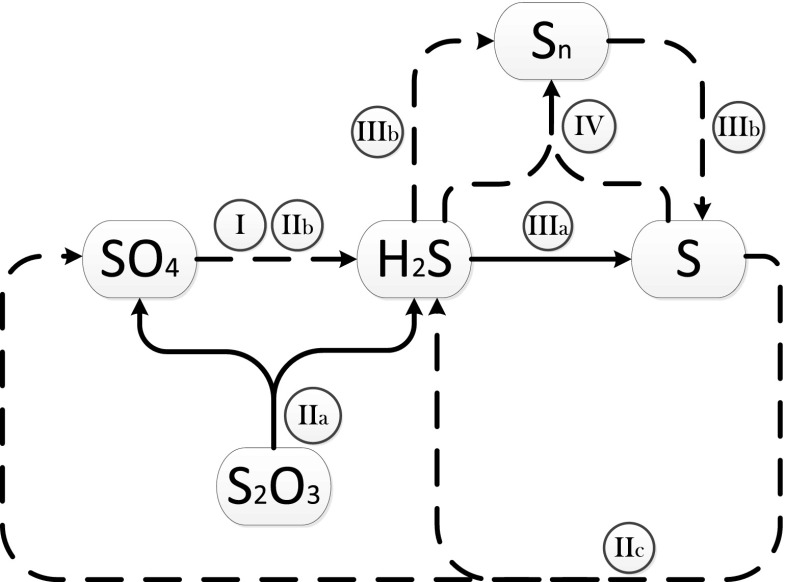
Sulphur compound reactions (possibly) occurring in the reactor. The *continuous lines* are conversions that occurred in the reactor. The *dotted lines* are some of the conversions that, from the pyrosequencing results, theoretically could have occurred, but no evidence was found. *I* Conversions made by sulphate reducers. *II Desulfocapsa* conversions; *IIa* was demonstrated; *IIb* might occur when short-chain alcohols are available; *IIc* might occur when the sulphide concentration is zero (Janssen et al. 1996; Finster et al. 1998). *III* The GSB conversions; sulphide might have been oxidized to sulphur directly (*IIIa*) or via polysulphide formation (*IIIb*) (Frigaard and Dahl 2009). However, polysulphide might also have been formed from the equilibrium between sulphur and sulphide (*IV*)

In the last phase (days 385–454) (phase B, Fig. 7), the reactor was completely covered with isolation foil to ensure complete darkness. As could be predicted, the complete lack of light affected the GSB metabolism (reaction 6). The percentage of sulphide produced that is related to the disproportionated thiosulphate was raised from 26 to 44 % (Table 4). However, the sulphide production did not match the thiosulphate disproportionation. It could be hypothesized that acetate was able to support the growth of GSB in the dark (Tang et al. 2011). However, it was completely depleted in the reactor when it was covered. Nevertheless, even though the net amount of produced sulphide did not increase as much as expected, the concentration of sulphide in the liquid phase did double (data no shown), providing another confirmation that the green sulphur bacteria were affected by complete lack of light. A summary of the reactions that occurred in the reactor is presented (Fig. 8).

It is relevant to realize that it is likely that in periods without mixing, different sections of the reactor might have been exposed to different light intensities, thus resulting in varying microbial activities which could concurrently have led to concentration, redox and microbial community gradients. However, as the reactor content was thoroughly mixed before sampling, the samples used for the different analyses are considered homogeneous and representative of the average composition/concentration present in the reactor.

To our knowledge, only one more reactor study dealing with thiosulphate disproportionation has been reported previously (Pikaar et al. 2013). Like in our study, they used a submerged membrane bioreactor system. However, they obtained higher thiosulphate disproportionation rates (8.4 vs. 0.79 mmol L^−1^ day^−1^ in our reactor) using granular and thermophilic biomass operating at 65 °C. Our system operates at a lower temperature, and it allows direct conversion to elemental sulphur. Both of these characteristics make the sulphur recovery easier and the process costs lower. Nevertheless, more research would be required if industrially relevant thiosulphate conversion rates need to be obtained.

## Conclusions

A bioreactor in which rates of anaerobic methane oxidation were increased was not obtained in this study. However, we obtained a system in which thiosulphate disproportionation and sulphide oxidation yielded sulphate, sulphide and sulphur out of thiosulphate. This process could be used to recover solid sulphur from thiosulphate. This thiosulphate could be generated from photographic fixing, pulp bleaching, gold leaching or fracking processes. However, additional studies should be performed to optimize this conversion by GSB. Such process would potentially allow cost reduction as other technologies used for this conversion rely in the use of additional reactors and raw materials such as air and/or metallic compounds.

## Electronic supplementary material

ESM 1 (docx 22 kb)
