# Supplementary material for: Thiosulphate conversion in a methane and acetate fed membrane bioreactor
Source: Environ Sci Pollut Res Int. 2015 Oct 1;23:2467–78. doi: 10.1007/s11356-015-5344-3 (PMC4717173; doi:10.1007/s11356-015-5344-3)
Supplement: Supplementary file 1 — (docx 22 kb) [file 11356_2015_5344_MOESM1_ESM.docx]

| **Primer** | **Primer sequence (5’-3’)** | **Reference** |
| --- | --- | --- |
| Adapter A | CCATCTCATCCCTGCGTGTCTCCGACTCAG | Provided by GATC Biotech |
| Adapter B | CCTATCCCCTGTGTGCCTTGGCAGTCTCAG | Provided by GATC Biotech |
| 27F-DegS | GTTYGATYMTGGCTCAG | Van den Bogert *et al*., 2011 |
| 338R-I | GCWGCCTCCCGTAGGAGT | Daims *et al*., 1999 |
| 338R-II | GCWGCCACCCGTAGGTGT | Daims *et al*., 1999 |
| A109f | ACKGCTCAGTAACACGT | Grosskopf *et al.,* 1998 |
| 1492R | GYTACCTTGTTACGACTT | Lane 1991 |

**SUPPLEMENTARY TABLES**

**Table S1** Primers used in this study for pyrosequencing and cloning of bacterial and archaeal 16S rRNA gene fragments, respectively.

| **Target** | **Primer name** | **Sequence (5’-3’)** | **Annaeling temperature (°C)** | **Extension time (s)** | **Amplicon size (bp)** | **Primer concentration (μM)** | Reference |
| --- | --- | --- | --- | --- | --- | --- | --- |
| *Archaea* | Arch-787f | ATTAGATACCCSBGTAGTCC | 55.4 | 40 | 272 | 0.4 | Yu *et al*., 2005 |
|  | Arch-1059r | GCCATGCACCWCCTC |  |  |  | 0.4 |  |
| ANME-2a | ANME2a-26F* | TGT TGG CTG TCC GGA TGA | 57.5 | 40 | 816 | 0.4 | Miyashita *et al.,*2009 |
|  |  | TGT TGG CTG TCC AGA TGA |  |  |  | 0.4 |  |
|  |  | TGT TGG CTG TCC AGA TGG |  |  |  | 0.4 |  |
|  | ANME2a-242R | AGG TGC CCA TTG TCC CAA |  |  |  | 0.4 |  |
| ANME-1 | ANME1-395F* | AAC TCT GAG TGC CTC CAA | 57.5 | 40 | 1022 | 0.4 | Miyashita *et al*., 2009 |
|  |  | AAC TCT GAG TGC CTC CTA |  |  |  | 0.4 |  |
|  |  | AAC TCT GAG TGC CCC CTA |  |  |  | 0.4 |  |
|  | ANME1-1417R* | CCT CAC CTA AAC CCC ACT |  |  |  | 0.4 |  |
|  |  | CCT CAC CTA AAT CCC ACT |  |  |  | 0.4 |  |

*These primers are a mixture of each of the stated primers inequimolar amounts, as described by Miyashita *et al*. 2009

**Table S2** Primers used for qPCR in this study with the corresponding annealing temperatures and concentrations

**REFERENCES**

- van den Bogert B., de Vos W.M., Zoetendal E.G., Kleerebezem M. 2011). Microarray analysis and barcoded pyrosequencing provide consistent microbial profiles depending on the source of human intestinal samples. Applied and Environmental Microbiology 77:2071-2080.
- Daims H., Bruhl A., Amann R., Schleifer K.H., Wagner M. 1999. The domain-specific probe EUB338 is insufficient for the detection of all Bacteria: Development and evaluation of a more comprehensive probe set. Systematic and Applied Microbiology. 22:434-444
- Grosskopf R, Janssen PH, Liesack W. 1998. Diversity and structure of the methanogenic community in anoxic rice paddy soil microcosms as examined by cultivation and direct 16S rRNA gene sequence retrieval. Appl. Environ. Microbiol. 64:960-969.
- Lane DJ. 1991. 16S/23S rRNA sequencing, p 115-175. In Stackebrandt E and Goodfellow M (ed), Nucleic acid techniques in bacterial systematics. Wiley & Sons, Chichester, United Kingdom.
- Miyashita A., Mochimaru H., Kazama H., Ohashi A., Yamaguchi T., Nunoura T., Horikoshi K., Takai K., Imachi H., 2009: Development of 16S rRNA gene-targeted primers for detection of archaeal anaerobic methanotrophs (ANMEs), FEMS Microbiology Letters. 297 (1): 31-37
- Yu Y, Lee C, Kim J, Hwang S. 2005. Group-specific primer and probe sets to detect methanogenic communities using quantitative real-time polymerase chain reaction. Biotechnology and Bioengineering. 89:670-679.
